# Bad Air

**Published:** 1869-01

**Authors:** 


### BAD AIR.

It is the great object of the Editor of this Journal to point out to the readers of it the many different methods by which they contract disease itself, or so impair their systems as to render them liable to "catch," as it is called, every epidemic which comes along. He believes it to be far better to avoid disease than to be obliged to cure it.

A fruitful source of disease, and a method of impairing the health of the laboring classes, is the practice the working people generally adopt of eating their dinners, or "lunches," in the rooms where they have been all the forenoon at work. In cold weather the air of their workshops is far more pernicious than in warm; is, in fact, almost pestilential, and to eat in this atmosphere, where the lungs are already full of it, is simply suicidal; and yet very many working people are doing this every day of their lives. Our business with printers and bookbinders renders it necessary for us to visit the places where they work almost daily, and to enter one of these offices in the latter part of the day, even for a stay of five minutes, so impregnates the lungs and clothing with this fetid air, that it requires a brisk walk in the open air to get rid of it. We advise every one, then, employed in a workshop or store, never to eat their meals there, at any rate, without first going out into the open air and walking or running off the foul air in the lungs and supplying them with fresh.

In all schools nearly, whether public or private, both teachers and scholars are a great portion of the time breathing an impure atmosphere. Many female teachers suffer from some trouble of the throat, and the scholars come home in the afternoon, as many a parent knows, with headache, and oftentimes nausea at the stomach, the legitimate consequences of breathing the fetid atmosphere of the school and class-rooms. Here, too, the practice prevails of allowing the pupils to eat their lunches at twelve o'clock, just after coming from the class-rooms, where they have been closeted for two or three hours, breathing contaminated atmosphere.

Some years since we were in the habit of visiting one of the large public schools of the city, and pointed out this mistake to the lady-principal, who was an exceedingly intelligent woman, and she promised to adopt the suggestion, to send out her pupils to the play-yard for ten minutes after they came from the class-room, and then call them in to eat their lunch. A week or so, subsequently, we visited the school again, and both teacher and scholars expressed themselves delighted with the change, and found themselves feeling much better for it, and they assured us that they should never return to their old ways.

We should never lose sight of the fact, that breathing foul air is one great source of disease, especially in the city, and we should exercise all the diligence we can command to avoid it, especially as a continuous breathing of bad air often lays the foundation for obscures disease which eventually rob life of all its joys and make it a failure.

---

We wish our readers and advertisers to bear in mind that we do not claim an interminable subscription list; but we believe that no other magazine published in this or any other country has as large a number of regular readers, larger for the present year than ever previously by many fold, as it goes to many libraries, reading-rooms and Christian Associations. We think it a reasonable estimate from the statistics which we have been enabled to gather, that this first number of the year, increasing as the months move on, will be read by members of seventy thousand different families, besides all private subscriptions; and this is the stimulus we shall ever have before our eyes, to write no line which shall not convey wholesome, useful and practical suggestions as to the conduct of life; and to aim, more than we have ever done before, to print no word that shall be against good morals, against religion, against the Bible, against the Sabbath-day, against woman, or against the ministers of the Christian system, but always in favor of all these, because upon them, and their high appreciation, all cultivated society, all successful and prosperous governments, all happy communities must stand.

